# Genetic and Functional Analysis of the Biosynthesis of a Non-Ribosomal Peptide Siderophore in *Burkholderia xenovorans* LB400

**DOI:** 10.1371/journal.pone.0151273

**Published:** 2016-03-10

**Authors:** María José Vargas-Straube, Beatriz Cámara, Mario Tello, Francisco Montero-Silva, Franco Cárdenas, Michael Seeger

**Affiliations:** 1 Laboratorio de Microbiología Molecular y Biotecnología Ambiental, Departamento de Química & Center for Nanotechnology and Systems Biology & Centro de Biotecnología, Universidad Técnica Federico Santa María, Valparaíso, Chile; 2 Centro de Biotecnología Acuícola, Departamento de Biología, Universidad de Santiago de Chile, Santiago, Chile; Belgian Nuclear Research Centre SCK•CEN, BELGIUM

## Abstract

*B*. *xenovorans* LB400 is a model bacterium for the study of the metabolism of aromatic compounds. The aim of this study was the genomic and functional characterization of a non-ribosomal peptide synthetase containing gene cluster that encodes a siderophore in *B*. *xenovorans* LB400. The *mba* gene cluster from strain LB400 encodes proteins involved in the biosynthesis and transport of a hydroxamate-type siderophore. Strain LB400 has a unique *mba* gene organization, although *mba* gene clusters have been observed in diverse *Burkholderiales*. Bioinformatic analysis revealed the presence of promoters in the *mba* gene cluster that strongly suggest regulation by the ferric uptake regulator protein (Fur) and by the alternative RNA polymerase extracytoplasmic function sigma factor MbaF. Reverse transcriptase PCR analyses showed the expression of iron-regulated transcriptional units *mbaFGHIJKL*, *mbaN*, *mbaABCE*, *mbaO*, *mbaP* and *mbaD* genes under iron limitation. Chrome azurol S (CAS) assay strongly suggests that strain LB400 synthesized a siderophore under iron limitation. Mass spectrometry ESI-MS and MALDI-TOF-MS analyses revealed that the siderophore is a non-ribosomal peptide, and forms an iron complex with a molecular mass of 676 Da. Based on bioinformatic prediction, CAS assay and MS analyses, we propose that the siderophore is L-*N*^*δ*^-hydroxy-*N*^*δ*^*-*formylOrn-D-β-hydroxyAsp-L-Ser-L-*N*^*δ*^-hydroxy-*N*^*δ*^-formylOrn-1,4-diaminobutane that is closely related to malleobactin-type siderophores reported in *B*. *thailandensis*.

## Introduction

Iron is essential for diverse metabolic processes in microorganisms. This element is involved in metabolic processes such as cellular respiration, Krebs cycle, oxygen transport, DNA synthesis, nitrogen fixation, photosynthesis, methanogenesis and hydrogen metabolism. Iron forms part of heme cofactors, iron-sulfur clusters and di-iron centers in proteins. In the presence of oxygen and at physiological pH, ferric ion is poorly soluble and therefore presents a rather low bioavailability. Aerobic microorganisms have developed iron-uptake systems which are required under iron-restricted conditions. Siderophores are low molecular mass compounds (<1000 Da) containing hydroxamate, *α*-hydroxycarboxylate and catechol ligands that covalently bind iron (III) with high affinity [1,2].

Diverse siderophores are composed of peptides assembled via non-ribosomal peptide synthetases (NRPS). They are composed of proteinogenic and non-proteinogenic amino acids that may be subjected to modification such as epimerization, glycosylation, acylation, methylation, formylation, halogenation, hydroxylation and cyclization [3]. NRPS catalyze the formation of peptide bonds between amino acid substrates by using biosynthetic modules composed of adenylation (A), thiolation (T), condensation (C) and epimerization (E) domains. The A domain is responsible for the activation of amino acid monomer into aminoacyl-AMP using ATP and for the catalysis of its transfer to the adjacent T domain to which monomer and nascent chain are bound. The C domain catalyzes the formation of the peptide bond between two adjacent modules, which leads to the elongation of the peptide [4]. The E domain catalyzes the epimerization from L to D form of the thioester-bound amino acid. These multimodular enzymes are governed by the colinearity rule [5], which is the basis for *in silico* prediction of the substrates that will be joined to each module and the structure of the final product. This rule is useful when searching for novel bioactive compounds synthesized by NRPS clusters [6,7].

The *Burkholderia* genus comprises both non-pathogenic environmental species and human pathogens such as *B*. *mallei* and *B*. *cepacia* [8–10]. In *Burkholderia*, NRPS systems for siderophore synthesis have been reported [11,12]. The siderophore ornibactin is synthesized by *B*. *vietnamiensis*, *B*. *cepacia*, *B*. *ambifaria* and *B*. *cenocepacia* [13,14], whereas malleobactin is produced by *B*. *pseudomallei*, *B*. *mallei* and *B*. *thailandensis* [15–17]. In some *Burkholderia* strains an additional siderophore has been reported, such as cepabactin in *B*. *cepacia* [13], pyochelin in *B*. *pseudomallei* [15], and cepaciachelin in *B*. *ambifaria* [12].

*B*. *xenovorans* LB400 is a model bacterium for the study of the degradation of a wide range of aromatic compounds including polychlorobiphenyls [18–25]. Recently, a functional polyhydroxyalkanoate anabolic pathway in strain LB400 has been reported [26]. The aim of this study was the genomic and functional characterization of a non-ribosomal peptide synthetase containing gene cluster that encodes proteins for the synthesis and transport of a siderophore in *B*. *xenovorans* LB400. Experimental analyses demonstrated that under iron limitation *B*. *xenovorans* LB400 produces a hydroxamate-type siderophore, which structure was elucidated by mass spectrometry.

## Materials and Methods

### *In silico* search and analysis of NRPS genes

A search for conserved domains of NRPS genes was performed in LB400 genome using the RPS-BLAST algorithm. The pfam00668 condensation domains provided by the Conserved Domains Database (CDD) (http://www.ncbi.nlm.nih.gov/cdd) were predicted. NRPS domain organization and binding pocket signatures of A domains were analyzed [5,27]. The Stachelhaus code is defined by 10 amino acid residues in the A domains that form the binding pocket of a specific substrate [27]. The substrate specificity prediction was carried out using the web-based program NRPS predictor2 (http://ab.inf.uni-tuebingen.de/toolbox/index.php?view=domainpred) [28]. Subsequently, this prediction was refined with available information in literature [16,17].

For the search of a siderophore gene cluster in other *Burkholderiales* strains, a BlastP search was accomplished using the translated NRPS sequence protein from the *BxeB0525* gene (MbaA) for translated sequence proteins from complete bacterial genomes of the NCBI Genome database, including the genomes of 43 *Burkholderiales* strains. 836 proteins hits were manually analyzed to identify genomes that have malleobactin- or ornibactin-type gene cluster. For the identification of iron regulated promoters in the siderophore gene cluster of LB400 strain, extracytoplasmatic function (ECF) sigma factor and ferric uptake regulator (Fur) binding sequences in the non-coding regions of the LB400 minor chromosome (C2) were searched (between the nucleotides 2.774.645 and 2.808.477), using the Artemis software (www.sanger.ac.uk/resources/software/artemis/). Sequence alignments were performed using the CLUSTALW algorithm [29].

The 3D structure of the MbaO protein was built using Phyre2 (www.sbg.bio.ic.ac.uk/phyre2/) to identify the structure homologous and obtain the alignment based on the secondary structure and Modeller (salilab.org/modeller/) to model the 3D structure. Ligand prediction was performed with 3Dligandsite (www.sbg.bio.ic.ac.uk/3dligandsite/). Transmembrane regions were analyzed by Sosui (harrier.nagahama-i-bio.ac.jp/sosui). As crystallized structures have not been reported, siderophore and ferrisiderophore 3D structures were predicted using Marvin Sketch (http://www.chemaxon.com). The binding site for siderophore and ferrisiderophore was predicted using a Docking strategy. Docking analyses between the 3D protein structure with the siderophore and ferrisiderophore were performed with Autodock 4 [30].

### Bacterial strain and culture conditions

For iron limitation assays, *B*. *xenovorans* LB400 was cultivated in MM9 mineral medium with trace solution supplemented with glucose (5 mM) as sole carbon and energy source at 30°C [22]. For iron-rich condition, bacterial cells were grown in MM9 mineral medium with trace solution supplemented with glucose (5 mM) and FeSO_4_ (45 μM). To study the effect of L-ornithine (Orn) on bacterial growth and siderophore activity under iron limitation, MM9 mineral medium with trace solution was supplemented with glucose (5 mM) and Orn (5 mM). Growth was determined by measuring turbidity at 600 nm.

### Siderophore activity

The presence of a siderophore was determined in LB400 culture supernatants by ferrisiderophore detection after incubation with FeCl_3_ (2 mM) for 30 min and measuring absorbance at 400 nm as well as by the chrome azurol S (CAS) colorimetric assay [31]. CAS activity was determined by the decrease in absorbance at 630 nm of the supernatant treated with CAS solution. Absorbance was measured after 2 h incubation at room temperature. Culture medium without cells treated with CAS solution was used as reference absorbance.

### RNA isolation and RT-PCR

*B*. *xenovorans* LB400 was grown on glucose until a turbidity at 600 nm of 0.5 (exponential phase) in presence of Orn (5 mM) or FeSO_4_ (45 μM). Total RNA was isolated from LB400 cells using an RNeasy mini kit (Qiagen, Hilden, Germany) according to the manufacturer´s recommendations. To degrade any residual DNA, DNase I treatment was carried out using the RNase-Free DNase Set (Qiagen, Hilden, Germany). Amplification of the 16S rRNA gene was used as control for DNA contamination using the 27f (5’-AGAGTTTGATCMTGGCTCAG-3’) and 1492r (5’-TACGGYTAC CTTGTTACGACTT-3’) primers [22]. RNA concentration was quantified using a QubitTM fluorometer (Invitrogen, Carlsbad, CA, USA). Reverse transcription-PCR (RT-PCR) was carried out using the Thermo Scientific Verso cDNA Kit according to the manufacturer’s instructions. Primers spanning intergenic regions of the *mba* genes were designed to evaluate the co-transcription of genes using RT-PCR. Sequence-specific primer pairs are listed in S1 Table. The cycles of amplification (30 cycles) were carried out after an initial denaturation at 94°C for 5 min as follows: 94°C for 1 min, 59°C for 1 min, 72°C for 2 min, and a final elongation at 72°C for 7 min. All primer pairs amplified fragments of the expected size from LB400 genomic DNA.

### Isolation of the ferrisiderophore complex

*B*. *xenovorans* LB400 was grown until late stationary phase (48 h) in the presence of Orn (5 mM) or FeSO_4_ (45 μM). Cultures (2 L) were centrifuged at 4,000 × *g* for 10 min at 4°C. The supernatant was concentrated to 200 mL under vacuum. Ferrisiderophore chelates were detected, by mixing the supernatant with FeCl_3_ (2 mM) for 30 min at room temperature, until the color of the supernatant turned yellow. The iron treated supernatant was centrifuged for 10 min, the siderophore fraction was separated on a Sep-Pak C8 column (Waters, Milford, MA, USA) and eluted with methanol (100 mL) to eliminate salts and concentrate the yellow colored fractions. Colored crude extracts were fractionated by several passes through a preparative HPLC using a RP-C18 column. Fractions were eluted using a solvent gradient (solvent A: water containing 0.1% TFA, solvent B: acetonitrile containing TFA 0.1%): 100% solvent A for 5 min, decreasing to 60% solvent A and 40% solvent B in 25 min, maintained at 100% solvent B for 5 min, followed by 100% solvent A for 5 min. Flow rate was kept at 3 mL min^-1^ at room temperature. Absorbance was monitored at 400 nm. Ferrisiderophore complexes of yellow fractions were characterized by analytical HPLC. A Jasco HPLC system equipped with a Chromolith Performance RP-18e column (100 x 4.6 mm) (Alltech Grom GmbH, Rottenburg, Germany) and a diode array detector module MD-2015 was used. Data processing was carried out with Jasco ChromPass software 1.7 (http://www.jasco.de/; Jasco Corporation, Tokyo, Japan). Ferrisiderophores were eluted at room temperature using a solvent gradient (solvent A: water containing 0.1% TFA, solvent B: acetonitrile containing TFA 0.1%): 100% solvent A for 2 min, decreasing to 60% solvent A and 40% solvent B in 6 min, maintained with 100% solvent B for 2 min and 100% solvent A for 2 min. Flow rates were kept at 1 mL min^-1^. Absorbance was monitored at 400 nm.

### Characterization of the ferrisiderophore complex by mass spectrometry

Ferrisiderophores complexes of yellow colored fractions were characterized by electrospray ionization (ESI-MS) and by matrix assisted laser desorption/ionization—time of flight (MALDI-TOF) mass spectrometry. For ESI-MS, samples prepared in a solvent containing acetonitrile 99.9% and TFA 0.1% were analyzed by Agilent 1100 HPLC system (Agilent Technologies, Santa Clara, CA, USA) coupled to an Esquire 4000 electrospray ion trap mass spectrometer (Bruker Daltonik, Bremen, Germany) as previously described [32]. As stationary phase, a C18 Luna column (5 μm particle size 100Å; 150 x 4.6 mm; Phenomenex, Torrance, CA, USA) connected to a split cell that divided the flow into the UV detector and into the mass spectrometer was used. As a solvent system, distilled water containing 0.1% formic acid (solvent A) and acetonitrile containing 0.085% formic acid (solvent B) were used. Ferrisiderophores were eluted using a solvent gradient from 3 to 53.3% solvent B within 30 min at room temperature. Flow rates were kept at 1 mL min^-1^. Absorbance was monitored at 400 nm. The ionization process by electrospray was performed at 4000 V, assisted by nitrogen as nebulizer gas, at a temperature of 325°C, pressure of 30 psi and a flow rate of 7.5 L/min. Mass spectra were acquired in positive polarity. The samples showed the corresponding [M(Fe)+H]^+^ ion as the ionized molecule of the ferrisiderophore complex.

For MALDI-TOF, samples containing ferrisiderophore were mixed with a *α*-cyano-4-hydroxycinnamic acid (CHCA 10 mg/mL in acetonitrile/formic acid 0.1% v/v in 1:1 ratio) matrix in a 1:1 ratio, and the mixture (2 μL) was deposited on a micro sample holder plate scout (Bruker Daltonics, Billerica, MA, USA). The peptide extracts were analyzed with a MALDI-TOF Microflex apparatus (Bruker Daltonics, Billerica, MA, USA) in positive ion mode using reflection detection (Centro de Estudio para el Desarrollo de la Química, Universidad de Chile, Santiago, Chile). The FlexControl 3.0 program (Bruker Daltonik, Bremen, Germany) was used to control the spectrometer. A calibration was performed with external standards: a mixture of peptides of 1000–3000 Da mass (Bruker Daltonics, Billerica, MA, USA). Spectra corresponds to the sum of 10 sweeps of 30 laser impacts applied at different points randomly taken from each sample deposited on the plate holder sample. The mMass program version 3.0 (http://www.mmass.org) was used to visualize the mass spectra.

## Results

**Fig 1:**
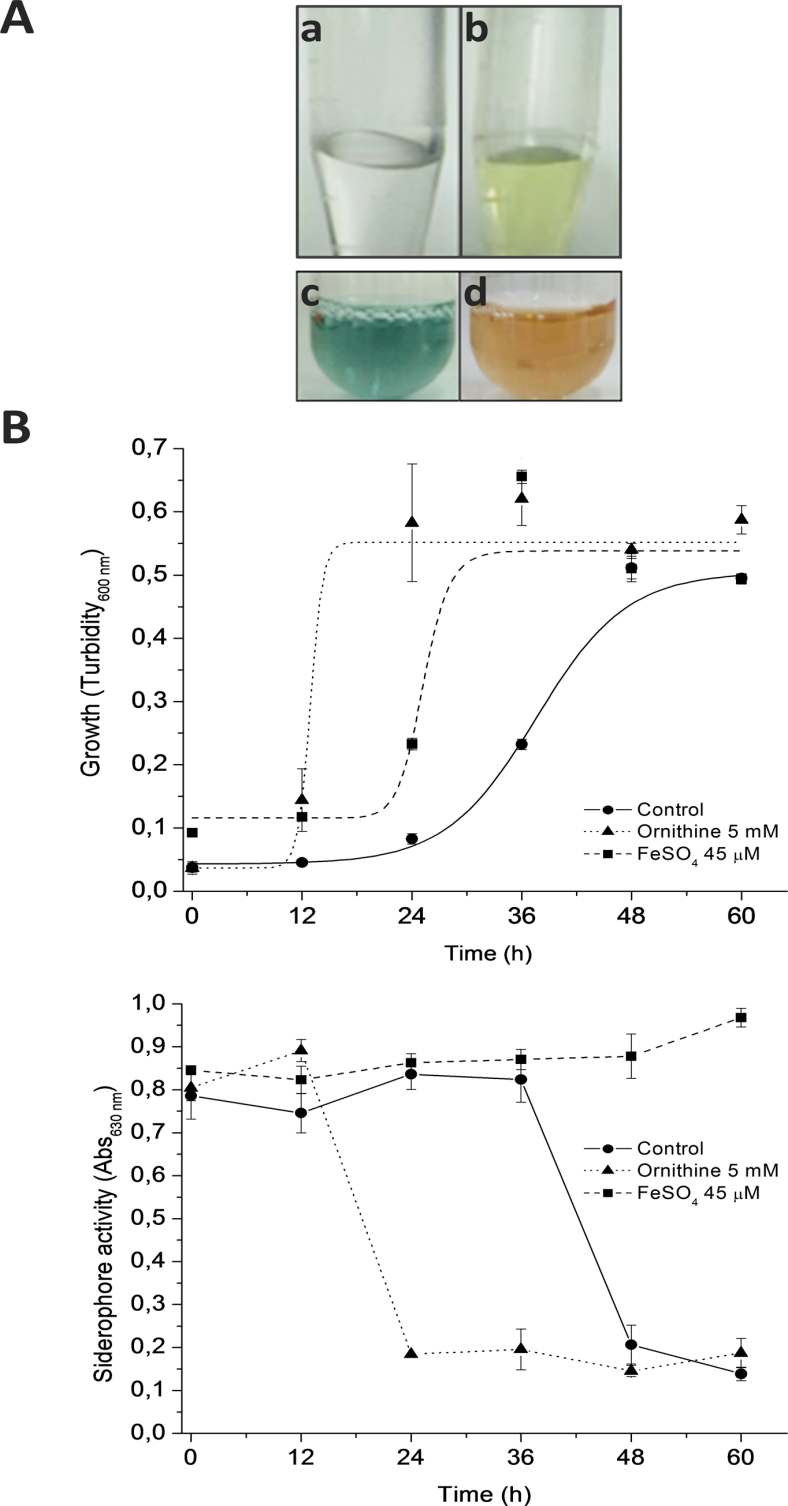
Siderophore synthesis under iron limitation in *B*. *xenovorans* LB400. A, Siderophore activity determined in supernatants of LB400 cells grown under iron-rich condition (a) and under iron limitation (b) and by the CAS assay of supernatants of LB400 cells grown under iron-rich condition (c) and under iron limitation (d). B, Effect of ornithine on LB400 growth and siderophore activity under iron limitation. LB400 cells were grown under iron-limitation in absence (control) or presence of ornithine (ornithine 5 mM), or under iron-rich condition (FeSO_4_ 45 μM).

In this report, the production of a siderophore by *B*. *xenovorans* LB400 was studied. In a first approach for ferrisiderophore complex detection, the supernatants of LB400 cells grown under iron limitation and iron-rich condition were incubated with FeCl_3_. The supernatant of cells grown under iron limitation and incubated with FeCl_3_ turned yellow (Fig 1A), indicating the formation of a ferrisiderophore complex, whereas the supernatant of cells grown in iron-rich condition remained colorless. In a second approach, the supernatants of LB400 cells were analyzed by the CAS colorimetric assay. The color change from blue to orange was observed only in the supernatant of LB400 cells grown under iron-limitation (Fig 1A), suggesting the presence of a siderophore. In contrast, the LB400 supernatant from iron-rich condition maintained the blue color. When the iron-limited medium was supplemented with ornithine, which is a non-proteinogenic amino acid used for the synthesis of diverse peptidic siderophores [3], LB400 cells reached earlier the stationary phase and a siderophore activity was observed after a shorter incubation time (Fig 1B). Fig 1 illustrates that in stationary phase, LB400 supernatants showed a higher siderophore activity than in exponential phase.

### Identification of a gene cluster involved in siderophore synthesis in *B*. *xenovorans* LB400

**Fig 2:**
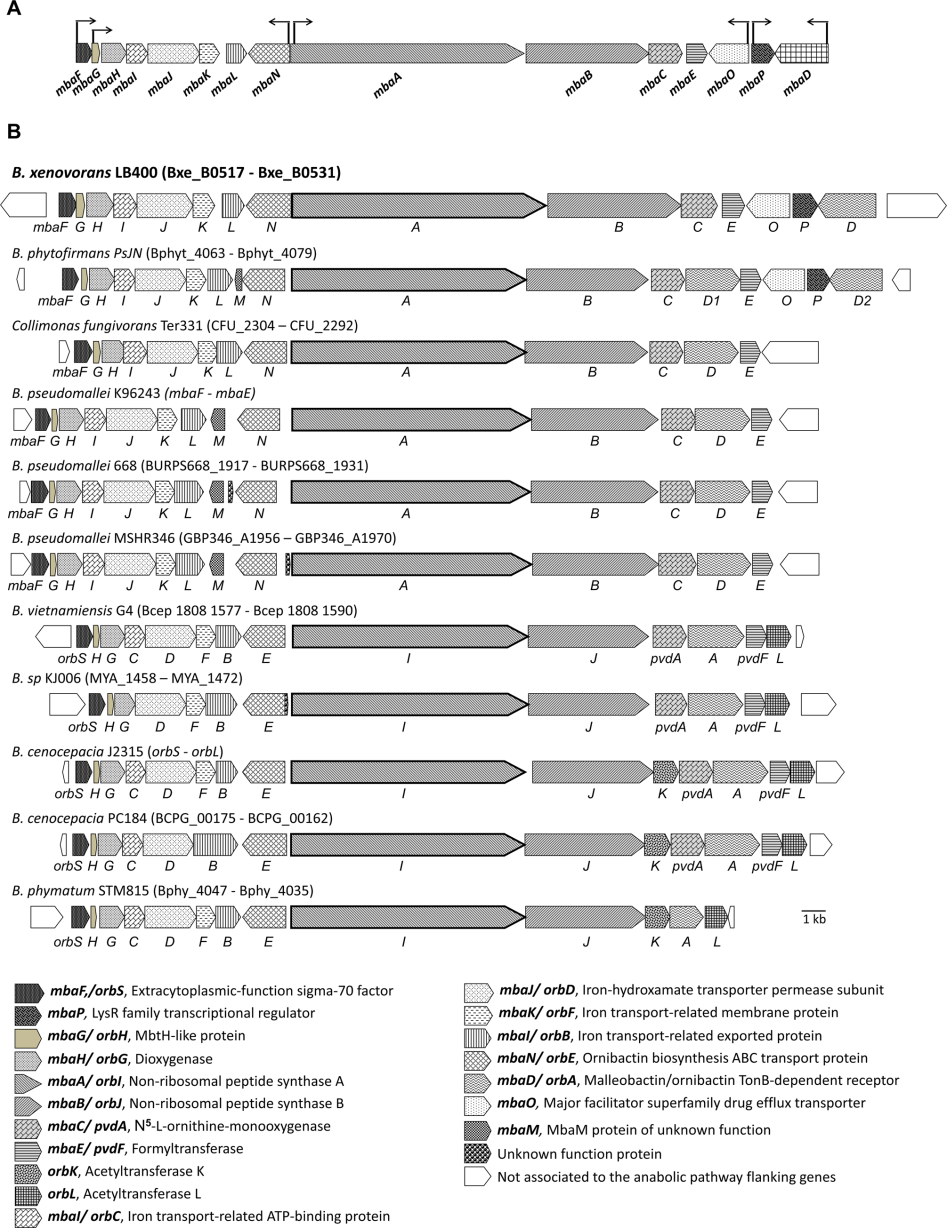
Organization of the *mba* gene cluster involved in the biosynthesis of siderophore in *B*. *xenovorans* LB400. A, Genetic organization of the *mba* genes located at the minor chromosome (C2). Genes are represented with block arrows. Sizes of the genes and the intergenic regions are on scale. The transcriptional promoters are represented with black arrows oriented in the transcription sense. B, Genetic organization of the *mba* (*orb*) genes in *Burkholderiales* strains. The *mba* (*orb*) sequences (accession number) are *B*. *xenovorans* LB400 (*BxeB0517*–*BxeB0531*), *B*. *phytofirmans* PsJN (Bphyt_4063–Bphyt_4079), *C*. *fungivorans* Ter331 (CFU_2304–CFU_2292), *B*. *pseudomallei* K96243 (*mbaF*–*mbaE*), *B*. *pseudomallei* 668 (BURPS6681917–BURPS668 1931), *B*. *pseudomallei* MSHR346 (GBP346_A1956–GBP346_A1970), *B*. *vietnamiensis* G4 (Bcep1808 1577–Bcep1808 1590), *Burkholderia* sp. KJ006 (MYA_1458–MYA_1471), *B*. *cenocepacia* J2315 (*orbS*–*orbL*), *B*. *cenocepacia* PC184 (BCPG00175–BCPG00162) and *B*. *phymatum* STM815 (Bphy4047–Bphy4035). The gene organization of *B*. *pseudomallei* K96243 is shared by *B*. *mallei* SAVP1 (BMASAVP1A_1634–BMASAVP1A _1621), *B*. *mallei* NCTC 10229 (BMA10229_A0295–BMA10229_A0284), *B*. *mallei* NCTC 10247 (BMA10247_0864–BMA10247_0877), *B*. *thailandesis* E264 (BTH I2427–BTH I2414) and *B*. *pseudomallei* 1710b (BURPS1710b_2076–BURPS1710b_2092). The gene organization of *B*. *pseudomallei* 668 is shared by *B*. *pseudomallei* 1106a (BURPS1106A 1931–BURPS1106A 1917) and *B*. *pseudomallei* BPC006 (BPC006_I1984–BPC006_I1999). The gene organization of *B*. *cenocepacia* J2315 is shared by *B*. *ambifaria* MC40-6 (BamMC406_1550–BamMC406_1564), *B*. *cepacia* AMMD (Bamb 1529–Bamb 1543), *B*. *cenocepacia* AU1054 (Bcen 1152–Bcen 1166), *B*. *cenocepacia* HI2424 (Bcen2424 1632–Bcen2424 1646), *Burkholderia* sp. 383 (Bcep18194 A4778–Bcep18194 A4792), *B*. *multivorans* ATCC17616 (Bmul 1606–Bmul 1593) and *B*. *cepacia* GG4 (GEM_1784–GEM_1770).

**Fig 3:**
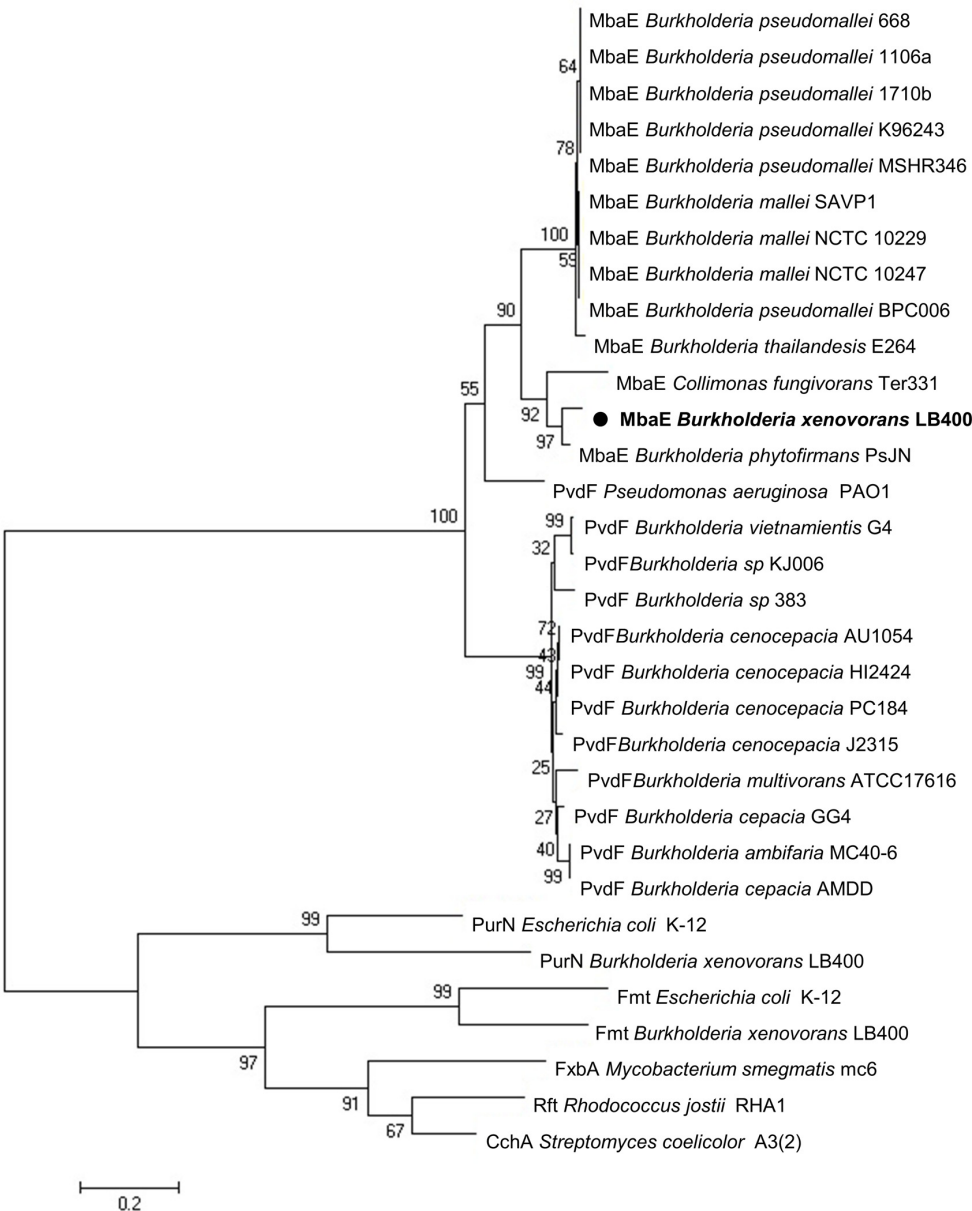
Phylogenetic analyses of MbaE formyltransferases. The dendogram of MbaE formyltransferase from *B*. *xenovorans* LB400 and other formyltransferases was constructed by the neighbor-joining method using MEGA 6.0 based on multiple sequence alignment by Clustal W. Sequence of deduced protein from *mbaE* gene from *B*. *xenovorans* LB400 is highlighted (black circle). One clade includes the L-*N*^δ-^hydroxy- ornithine formyltransferases (accession number) that may produce L-*N*^δ^-hydroxy-*N*^δ^-formylornithine and L-*N*^δ^-hydroxy-*N*^α^-formylornithine: MbaE *B*. *xenovorans* LB400 (YP554769.1), MbaE *B*. *phytofirmans* PsJN (YP001887830.1), MbaE *C*. *fungivorans* Ter331 (YP004752942.1), MbaE *B*. *pseudomallei* 668 (YP001058966.1), MbaE *B*. *pseudomallei* 1106a (YP001066211.1), MbaE *B*. *pseudomallei* 1710b (YP333487.1), MbaE *B*. *pseudomallei* K96243 (YP108374.1), MbaE *B*. *pseudomallei* MSHR345 (YP002896675.1), MbaE *B*. *mallei* SAVP1 (YP992947.1), MbaE *B*. *mallei* NCTC 10229 (YP001026286.1), MbaE *B*. *mallei* NCTC 10247 (YP001080440.1), MbaE *B*. *pseudomallei* BPC006 (YP006652779.1) and MbaE *B*. *thailandesis* E264 (YP442935.1). The other clade includes L-*N*^δ^-hydroxy-ornithine formyltransferases (accession number) that produce L-*N*^δ^-hydroxy-*N*^δ^-formylornithine: PvdF *P*. *aeruginosa* PA01 (NP251086.1), PvdF *B*. *vietnamientis* G4 (YP001119431.1), PvdF *Burkholderia sp*. KJ006 (YP006332563.1), PvdF *Burkholderia sp*. 383 (YP369030.1), PvdF *B*. *cenocepacia* AU1054 (YP621045.1), PvdF *B*. *cenocepacia* HI2424 (YP835290.1), PvdF *B*. *cenocepacia* PC184 (YP002091473.1), PvdF *B*. *cenocepacia* J2315 (YP002230827.1), PvdF *B*. *multivorans* ATCC17616 (YP001579778.1), PvdF *B*. *cepacia* GG4 (YP006615890.1), PvdF *B*. *ambifaria* MC40-6 (YP001808266.1), PvdF *B*. *cepacia* AMDD (YP773434.1). The methionyl-tRNA_f_^Met^ formyltransferases (accession number) are Fmt *B*. *xenovorans* LB400 (YP556637.1) and Fmt *E*. *coli* K-12 (YP492145.1) and the glycinamide ribonucleotide formyltransferases (accession number) are PurN *B*. *xenovorans* LB400 (YP560177.1) and PurN *E*. *coli* K-12 (YP490728.1).

The pfam00668 protein domain that catalyzes the formation of a peptide bond through a condensation and is highly conserved in NRPS systems [33] was screened for in the LB400 genome. Five NRPS condensation domains were found to belong to two NRPS encoded by the *mbaA* (*BxeB0525*) and *mbaB* (*BxeB0526*) genes located on the LB400 chromosome 2 (Fig 2). A ~30 kb gene cluster with 15 coding sequences organized in operon-type structures that could be involved in the synthesis and transport of a hydroxamate-type siderophore was determined (Fig 2, Table 1). The cluster is flanked upstream by the *BxeB0516* gene, which encodes a lipoprotein, and downstream by the *BxeB0532* gene that encodes the NADP-dependent isocitrate dehydrogenase. The coding sequences involved in the siderophore synthesis are the *BxeB0519* gene and the *BxeB0525*-*BxeB0528* genes. The *BxeB0525* (hereafter *mbaA*) and *BxeB0526* (hereafter *mbaB*) genes encode two NRPS with high identity to MbaA (73%) and MbaB (72%) proteins that are involved in malleobactin synthesis in *B*. *pseudomallei* [15]. The predicted amino acid sequence of the *BxeB0527* (hereafter *mbaC*) gene shows high identity (74%) to Orn monooxygenases (MOs) MbaC from *B*. *pseudomallei* and PvdA (73%) from *B*. *cenocepacia* that are involved in *N*^*δ*^-hydroxylation of Orn [16,34]. The *BxeB0528* (hereafter *mbaE*) gene encodes a *N*^*δ*^-hydroxyornitine formyltransferase (FT) with 76% identity to PvdF from *Pseudomonas aeruginosa* PA01 [35]. Fig 3 illustrates a phylogenetic tree for MbaE FTsin that MbaE from strain LB400 clusters together with MbaE from *B*. *phytofirmans* PsJN and MbaE from *Collimonas fungivorans* Ter331. *BxeB0519* gene (hereafter *mbaH* gene) encodes an α-ketoglutarate-dependent hydroxylase with high identity (84%) to OrbG that is involved in ornibactin synthesis in *B*. *cenocepacia* [14]. *BxeB0518* gene (hereafter *mbaG* gene) encodes a MbtH-like protein that may participate as regulator in the siderophore synthesis. It has been proposed that MbtH-like proteins are regulators of NRPS, interacting with adenylating enzymes [36]. The coding sequences of the *mba* cluster probably involved in the transport and recognition of a hydroxamate ferrisiderophore complex are *BxeB0520–BxeB0524* genes, *BxeB0529* and *BxeB0531* genes. *BxeB0520* (hereafter *mbaI*), *BxeB0521* (hereafter *mbaJ*) and *BxeB0523* (hereafter *mbaL)* genes encode inner membrane, transmembrane and periplasmic transporter proteins from the ATP-binding cassette (ABC) family, which are involved in the uptake of hydroxamate-type ferrisiderophores [14,37,38]. The gene product of the *BxeB0522* (hereafter *mbaK*) gene has 55% identity to protein OrbF from *B*. *cenocepacia*. This protein is probably involved in the reduction of intracellular ferric iron from the internalized ferrisiderophore complex [14]. The *BxeB0524* (hereafter *mbaN*) gene encodes an ABC transporter protein with 82% identity to OrbE from *B*. *cenocepacia* [14]. MbaN is probably involved in the export of the siderophore through the cytoplasmic membrane. The *BxeB0529* (hereafter *mbaO*) gene encodes a major facilitator superfamily (MFS) transporter [25] that may be involved in siderophore transport. MbaO protein shows high identity to MFS transporters from *Burkholderia* strains such as *B*. *phytofirmans* PsJN (90% identity), *B*. *multivorans* ATCC 17616 (70%), *B*. *cenocepacia* PC184 (67%), and *B*. *cepacia* GG4 (67%), and shows 67% identity to the MFS drug efflux transporter EmrB/QacA from *Pseudomonas* sp. GM102 [39]. Modelling of the MbaO 3D structure indicates that the MFS transporter possesses 14 transmembrane regions, and docking analyses indicate potential binding sites for malleobactin and ferrimalleobactin located close to the MbaO cytoplasmic side (S1 Fig). The *BxeB0531* (hereafter *mbaD*) gene encodes an outer membrane TonB-dependent siderophore receptor that has 23% sequence identity to the ornibactin receptor OrbA from *B*. *cenocepacia* [40], and shows high conservation of TonB-dependent receptor domains. MbaD possess the TonB-dependent gated channel domain (cd01347) that permits the interaction with the ferrisiderophore complex, suggesting that MbaD is probably involved in the internalization of the ferrisiderophore. The *BxeB0517* (hereafter *mbaF*) gene encodes a RNA polymerase ECF sigma factor that has high identity with OrbS (71%) from *B*. *cenocepacia* [14] and MbaS (79%) from *B*. *pseudomallei* [15]. The *BxeB0530* (hereafter *mbaP*) gene encodes a transcriptional regulator of the LysR family that has 32% identity to the transcriptional regulator PtxR from the pyoverdin biosynthesis system in *P*. *aeruginosa* [41].

**Table 1:** Predicted genes encoding the siderophore biosynthesis pathway in *B*. *xenovorans* LB400.

ORF	Gene	aa	Related gene products		
			Protein function	Organism	% Id (aa)
BxeB0517	*mbaF*	223	extracytoplasmatic function sigma factor	*B*. *pseudomallei* K96243	79 (154/194)
BxeB0518	*mbaG*	105	MbtH-like protein	*B*. *cenocepacia* J2315	79 (61/77)
BxeB0519	*mbaH*	340	α-ketoglutarate dioxygenase	*B*. *cenocepacia* J2315	84 (275/329)
BxeB0520	*mbaI*	288	Fe^3+^/hydroxamate transporter inner membrane subunit	*B*. *cenocepacia* J2315	86 (214(248)
BxeB0521	*mbaJ*	704	Fe^3+^/hydroxamate transporter permease subunit	*B*. *cenocepacia* J2315	74 (512/695)
BxeB0522	*mbaK*	272	Iron reductase	*B*. *cenocepacia* J2315	55 (148/270)
BxeB0523	*mbaL*	276	Fe^3+^/hydroxamate transporter periplasmic component	*B*. *cenocepacia* J2315	67 (181/270)
BxeB0524	*mbaN*	562	ABC peptide transporter	*B*. *pseudomallei* K96243	88 (492/561)
BxeB0525	*mbaA*	3180	Non ribosomal peptide synthase NRPS I	*B*. *pseudomallei* K96243	73 (2378/3252)
BxeB0526	*mbaB*	1576	Non ribosomal peptide synthase NRPS J	*B*. *pseudomallei* K96243	72 (1244/1731)
BxeB0527	*mbaC*	456	*N*^δ^-L-ornithine-monooxygenase	*B*. *pseudomallei* K96243	74 (347/468)
BxeB0528	*mbaE*	280	Formyltransferase	*B*. *pseudomallei* K96243	81 (225/278)
BxeB0529	*mbaO*	543	MFS transporter	*Pseudomonas sp*. GM102	67 (395/503)
BxeB0530	*mbaP*	312	LysR-type transcriptional regulator	*P*. *aeruginosa* PAO1	32 (94/291)
BxeB0531	*mbaD*	727	TonB-dependent siderophore receptor	*B*. *pseudomallei* K96243	27 (194/718)

A bioinformatic search of additional NRPS-dependent and NRPS-independent biosynthetic genes involved in siderophore production in LB400 genome was performed. In the LB400 genome, genes from the pyochelin, cepabactin and cepaciachelin biosynthetic pathways were not found. Catalytic domains of enzymes involved in the biosynthesis of non-peptidic siderophores, such as aerobactin, desferroxyamin, alcalygin, antrachelin, acromobactin, estafilobactin, rhizobactin 1021 and vibrioferrin were also not detected in LB400 genome. In this study, the *mba* cluster was the only gene cluster involved in siderophore biosynthesis found in strain LB400 genome.

### Gene clusters involved in siderophore biosynthesis in *Burkholderiales*

The LB400 NRPS gene cluster shows high similarity with gene clusters that synthesize hydroxamate-type siderophores from other *Burkholderia* strains. To compare NRP siderophore gene cluster among *Burkholderia* genomes, a search of these genes in other *Burkholderiales* strains was performed. Eleven different gene clusters were identified in 24 *Burkholderia* strains and one *Collimonas* strain (Fig 2). These gene clusters differ in the presence and distribution of specific genes. In general, there are two types of gene clusters for the synthesis of siderophores belonging either to malleobactin or ornibactin-type compounds. The *mba* and *orb* gene clusters differ in the first A domain (A_1_) from NRPS that activates L-*N*^*δ*^-hydroxy-*N*^*δ*^-formylornithine (hfOrn) and L-*N*^*δ*^-hydroxy-*N*^*α*^-formylornithine (hαfOrn) in malleobactins and L-*N*^*δ*^-hydroxy-*N*^*δ*^-acylornithine (haOrn) in ornibactins. Another difference is the presence or absence of the *orbK* and *orbL* genes, which encode *N*-acetyltransferases probably involved in the acylation of L-*N*^*δ*^-hydroxyornithine (hOrn) in ornibactins [17,42–44]. On the one hand, *B*. *xenovorans* LB400, *B*, *vietnamensis* G4, *B*. *phytofirmans* PsJN, *C*. *fungivorans* Ter331 and *B*. *pseudomallei* strains K96243, 668 and MSHR346 *mba* gene clusters are involved in the synthesis of malleobactin-type siderophores that contain the first Orn of the tetrapeptide as hfOrn or hαfOrn. On the other hand, *Burkholderia* sp. KJ006, *B*. *cenocepacia* strains J2315 and PC184, and *B*. *phymatum* STM815 *orb* gene clusters are associated with the synthesis of ornibactin-type compounds that possess the first tetrapeptide Orn as haOrn. Interestingly, two novel coding sequences (*mbaO* and *mbaP*) located downstream of the biosynthesis genes were identified in the LB400 gene cluster (Fig 2). These genes are present in the gene cluster identified in this study in *B*. *phytofirmans* PsJN (Fig 2), but are not present in the functional *mba* and *orb* gene clusters from *B*. *cenocepacia* and *B*. *pseudomallei* [14,15].

### *In silico* analyses of promoter regions in strain LB400

To search for promoters, the non-coding regions upstream of each *mba* gene from LB400 strain were analyzed *in silico*. Bioinformatic analyses suggest the presence of different operons in the *mba* gene cluster of strain LB400. Six potential iron-regulated promoters were identified. Promoter regions of *mbaG*, *mbaN*, *mbaA* and *mbaP* genes contain sequences with high identity to the consensus sequence ^G^_C_G^G^_C_TAAA^A^_T_A^A^_T_^A^_T_N(^G^_C_)_9_NNNCGTC that includes the -10 and -35 elements for the promoter recognition of the RNA polymerase sigma factor OrbS for ornibactin synthesis in *B*. *cenocepacia* [14]. The *mbaG*, *mbaN*, *mbaA* and *mbaP* gene promoter sequences show a match of 25/28, 26/28, 25/28 and 20/28 with the consensus OrbS binding sequence, respectively. The RNA polymerase ECF sigma factor OrbS in *B*. *cenocepacia* and PvdS in *P*. *aeruginosa* (MbaF in *B*. *xenovorans* LB400) activates the expression of genes for the ornibactin and pyoverdin synthesis, respectively, when the ferric uptake regulator protein Fur is not exercising its repressor activity [14,45]. Strain LB400 possesses a *fur* gene (*BxeA0571*) that probably encodes a ferric uptake regulator protein. LB400 Fur protein shows 56% sequence identity with the Fur regulator from *P*. *aeruginosa* [46]. The promoter regions of the *mbaF* and *mbaD* genes were found to contain sequences with high identity to the Fur box (GTAAACGCAAATCATTCTC) in *B*. *cenocepacia* [14]. The Fur box of *mbaF* and *mbaD* genes promoters show a match of 17/19 and 12/19 with the *fur* sequence of the *orbS* gene from *B*. *cenocepacia*. It has been reported that a match of 13/19 with the Fur binding sequence (GATAATGATAATCATTATC) from *E*. *coli* [45] may constitute a functional Fur box in the *orbS* gene promoter [14]. The presence of Fur-binding elements in the promoters of *mbaF* and *mbaD* genes suggests the regulation of their expression by the Fur protein. Two potential rho-independent terminators transcription sequences were identified in the *mba* gene cluster from strain LB400. One terminator is located 2343 nt upstream from the predictive initiation codon of the *mbaO gene* and the other is located 3417 nt upstream from the predictive initiation codon of the *mbaP* gene. The *mbaP* gene promoter possesses a probably Fur box and also a potential LysR-type transcriptional regulator binding site (5’-TCGGCGTCCCTA-3´ sequence that is at -57 from the *mbaP* gene) for its autoregulation. The divergent orientation of transcription of the *mbaP* and *mbaO* genes, the presence of a LysR-type transcriptional regulator binding site (5’-TCCGATCGCACGGA-3´ sequence at -57 from the *mbaO* gene) and the absence of Fur and MbaF promoter binding elements upstream of the *mbaO* gene suggest that its transcription is activated by the MbaP regulator.

### Transcriptional analysis of the *mba* gene cluster

**Fig 4:**
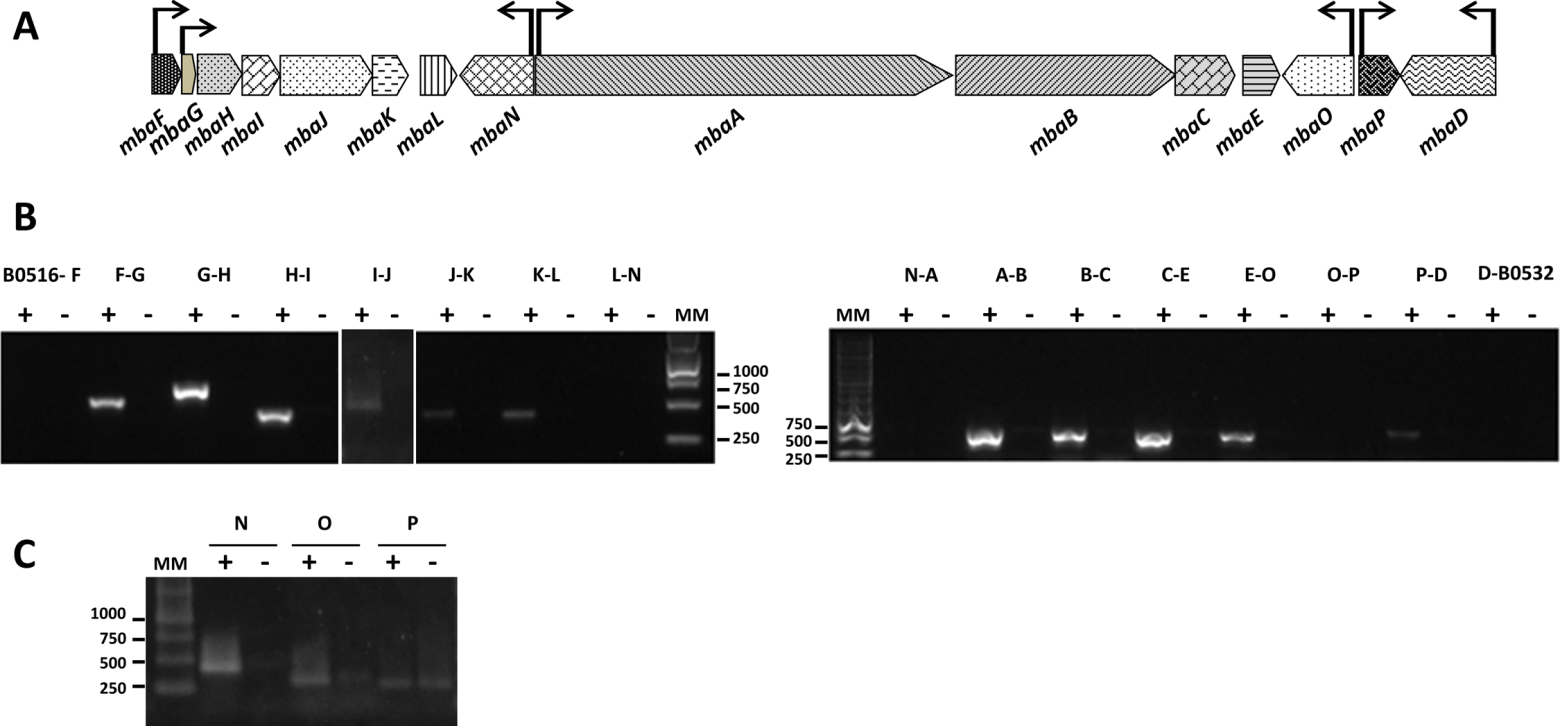
Expression of the *mba* genes from *B*. *xenovorans* LB400. A, Gene organization of the *mba* operon. Genes are represented with block arrows indicating gene orientation. Proposed biosynthetic genes are shown in dark gray, transport and utilization genes in light gray, and regulation genes in black. Transcriptional units predicted from RT-PCR analyses are shown by horizontal line arrows, with the promoter regions depicted by the start of the arrows. B, RT-PCR analysis of transcripts from the intergenic regions of the *mba* gene cluster. C, RT-PCR analysis of the expression of the *mbaN*, *mbaO* and *mbaP* genes. Amplification products were subjected to electrophoresis in an agarose (1%) gel. MM, molecular size markers (1 kb and Express DNA ladder). Expression of each region was analyzed under iron-limitation (+) and without iron limitation (-).

To evaluate if the *mba* genes from LB400 are functional, expression of the *mba* gene cluster in strain LB400 was evaluated under iron limitation and iron-rich condition. Under iron limitation, transcription products were observed for intergenic regions *mbaFGHIJKL and mbaABCE*, and for the *mbaN*, *mbaO* and *mbaP* genes. In contrast, cells grown in a medium supplemented with iron only expressed *mbaO* and *mbaP* genes, suggesting that the *mba* cluster transcription in strain LB400 is regulated by iron (Fig 4). Intergenic regions between *BxeB0516*-*mbaF*, *mbaN-mbaA*, *mbaO-mbaP* and *mbaD-BxeB0532* were not transcribed since adjacent genes are orientated in opposite directions. Although both *mbaE-mbaO* and *mbaP-mbaD* genes are orientated in opposite directions, these intergenic regions were expressed under iron limitation. For both cases, rho-independent terminator transcription sequences were identified. Co-expression of intergenic regions and the bioinformatic analysis of the promoters, strongly suggests the presence of the transcriptional units *mbaFGHIJKL*, *mbaN*, *mbaABCE*, *mbaO*, *mbaP* and *mbaD* genes in the *mba* cluster from *B*. *xenovorans* LB400 (Fig 4).

### Prediction of the siderophore structure

**Fig 5:**
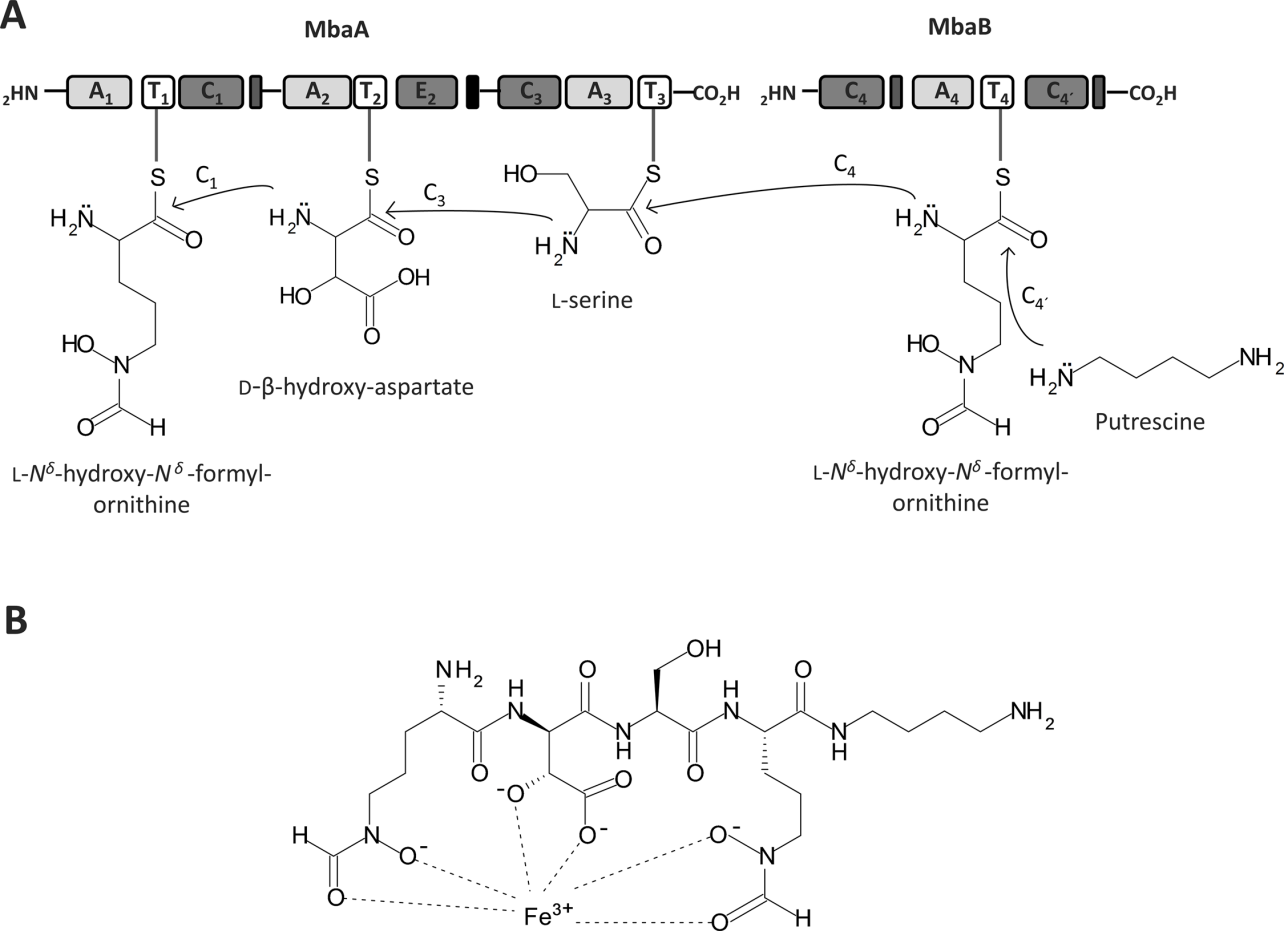
Siderophore synthesis by the non-ribosomal peptide synthetases MbaA and MbaB in *B*. *xenovorans* LB400 and the ferrisiderophore complex structure. A, Modular organization of the non-ribosomal peptide synthetases encoded by the *mbaA* and *mbaB* genes. Domains are represented as blocks. Adenylation (A), thiolation (T), condensation (C) and epimerization (E) domains of MbaA are organized in three modules. Domains of MbaB are organized in one module. The synthesis of the siderophore starts with specific substrates assembly to each NRPS module. Substrate of modules 1 and 4 is L-*N*^δ^-hydroxy-*N*^δ^-formylornithine (hfOrn). Substrates of module 2 and 3 are D-β-hydroxy-aspartic acid (OH-Asp) and L-serine (Ser), respectively. Condensation domains of each module allow the attachment of the four substrates to form the tetrapeptide. C_1_ condenses hfOrn with OH-Asp, C_3_ condenses OH-Asp with Ser and C_4_ condenses Ser with hfOrn. Chemical modifications of the amino acids are catalyzed by the protein products of *mbaH*, *mbaC* and *mbaE* genes before the assembly to each NRPS module. The α-ketoglutarate-dependent hydroxylase MbaH hydroxylates the β-carbon of Asp. The L-ornithine-5-monooxygenase encoded by the *mbaC* gene hydroxylates the nitrogen δ of both Orn. The formyltranserase encoded by the *mbaE* gene formylates the nitrogen δ of Orn 1 and 4. C_4´_ domain of module 4 adds a diaminobutane (putrescine) molecule in the N-terminus of the modified tetrapeptide. B, Ferrisiderophore complex structure synthesized by *B*. *xenovorans* LB400. The ferrisiderophore structure was predicted by bioinformatic analyses and confirmed by ESI-MS and MALDI-TOF MS of the complex purified by HPLC.

The substrate prediction for the NRPS enzymes encoded by genes *mbaA* and *mbaB* from *B*. *xenovorans* LB400 and the comparison of bacterial *mba* and *orb* gene clusters allowed the *in silico* prediction of the siderophore structure. Proteins encoded by *mbaA* and *mbaB* genes from strain LB400 have conserved NRPS domains that show high identity to NRPS domains for malleobactin and ornibactin synthesis in *B*. *pseudomallei* and *B*. *cenocepacia*, respectively [14–16,42]. MbaA has A, T, C and E domains organized in three biosynthetic modules (modules 1, 2 and 3). MbaB presents A, T and C domains organized in one module (module 4) as well as a second C domain (Fig 5). A prediction of the specific substrates for each module was carried out based on amino acid code of Stachelhaus of the A domains and information available in literature (Table 1). MbaA module 1 is predicted to use as substrate hOrn, hfOrn, hαfOrn or Phe based on previous reports [16,17]. For MbaA module 2 the use of L-β-hydroxy-aspartic acid (hAsp) as substrate is proposed [16]. Three conserved residues (Thr239, Lys278 and His322) that interact with hAsp are present in the second A domain [27,47]. Substrate prediction for MbaA module 3 indicates the use of L-serine (Ser). The catalytic dyad His278 and Ser301 that interacts with a Ser is present in the third A domain [27,47]. MbaB module 4 shows specificity for hfOrn (Table 2). The substrates Orn and Asp probably undergo tailoring reactions before assembling to its corresponding module. Asp is probably hydroxylated at the β-carbon by the α-ketoglutarate-dependent hydroxylase MbaH and epimerized to D-β-hydroxy-aspartic acid by the E_2_ domain of MbaA module 2. Orn for modules 1 and 4 is probably first hydroxylated by MO MbaC and subsequently formylated by FT MbaE. The fourth Orn attached to MbaB may be subjected to an additional modification that is an amidation at the C-terminus with the addition of 1,4-diaminobutane (putrescine) by the C_4´_ domain from MbaB module 4 (Fig 5). The Stachelhaus-code of the predicted Orn activation domains of modules 1 and 4 in strain LB400 show the universally conserved residues Asp235 and Lys517 (in all amino acid activating domains), as well as the Orn-specific hydrophobic residues Gly301 and Ile330 [47]. However, the presence in all Orn activation domains in *Burkholderiales* strains of Glu239 instead of Glu278 that is present in gramicidin, tyrocidine, fengycin and bacitracin synthetase Orn A domains, suggests its interaction with oxidized forms of Orn. This Glu position change, which also has been observed in the first A domain of exochelin synthetase FxbC (A_1__FxbC) involved in the activation of hOrn [47–49], is probably a feature of domains that activate an oxidized Orn. Hydroxylation occurs at nitrogen δ in both Orn 1 and Orn 4 of malleobactin, producing hOrn. However, contrasting with ornibactin, the malleobactin molecule can undergo formylation at nitrogen δ and α of hOrn 1 [17]. On the other hand, phylogenetic analysis of FTs MbaE in *Burkholderiales* strains suggests that MbaE of strain LB400 is closely related to MbaE from *B*. *thailandensis* that is able to formylate nitrogen δ and α from the first hOrn and nitrogen δ from the hOrn 4 in malleobactins [17], whereas FT PvdF is able to formylate only nitrogen δ of both hOrn in ornibactin and pyoverdin. FTs MbaE and PvdF belong to markedly different clades in the dendogram (Fig 3). Incubation with ferric iron and CAS assay indicate that strain LB400 produces a malleobactin siderophore that forms a stable ferrisiderophore complex, suggesting that the first hOrn of the LB400 malleobactin is formylated at nitrogen δ generating a hydroxamate group that provides one of the predicted Fe-binding sites. Franke *et al*. proposed that malleobactin E is the only malleobactin of *B*. *thailandensis* E264 that possesses siderophore activity due to its three Fe-binding sites including the hydroxamate group at Orn 1 [17]. Therefore, the predicted chemical structure of the active siderophore synthesized by the Mba NRPS system of strain LB400 is the linear tetrapeptide hfOrn-D-β-hydroxyAsp-Ser-hfOrn-1,4-diaminobutane (Fig 5). The molecule has three functional hydroxamate groups that coordinate Fe^3+^ with three bidentate bonds, forming the ferrisiderophore complex. The predicted molecular mass for the ferritetrapeptide complex matches 675.2037 Da, with a siderophore: iron stoichiometry of 1:1 (Fig 5).

**Table 2:** Prediction of MbaA and MbaB adenylation domains specificities in *B*. *xenovorans* LB400 based on literature [16,17,27].

	Stachelhaus-code residues										Predicted substrate
A-Domain	235	236	239	278	299	301	322	330	331	517	
A_1_	D	V	E	T	L	G	G	I	S	K	hfOrn/hαfOrn/hOrn/Phe
A_4_	D	G	E	Y	T	G	G	I	T	K	hfOrn
A_2_	D	L	T	K	V	G	H	V	G	K	β-OH-Asp
A_3_	D	V	W	H	V	S	L	I	D	K	Ser

### Structure analyses of the siderophore

**Fig 6:**
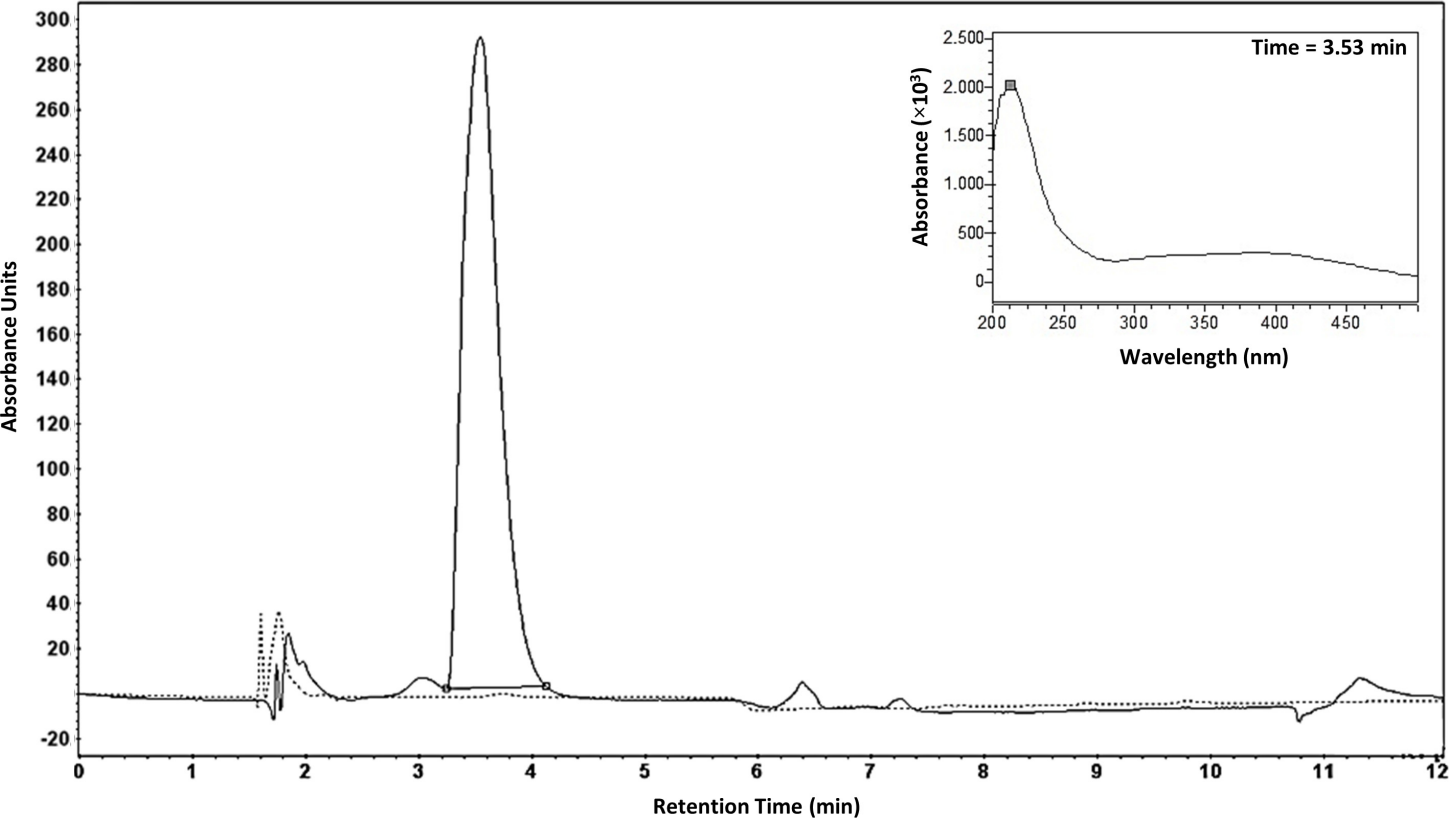
Purification of the ferrisiderophore complex from *B*. *xenovorans* LB400. HPLC chromatogram of the supernatant of LB400 cells in an iron-depleted (black line) and an iron-supplemented (dotted line) culture. The peak with retention time of 3.53 min contains the ferrisiderophore complex. The absorbance spectrum of the ferrisiderophore complex is depicted in the insert.

**Fig 7:**
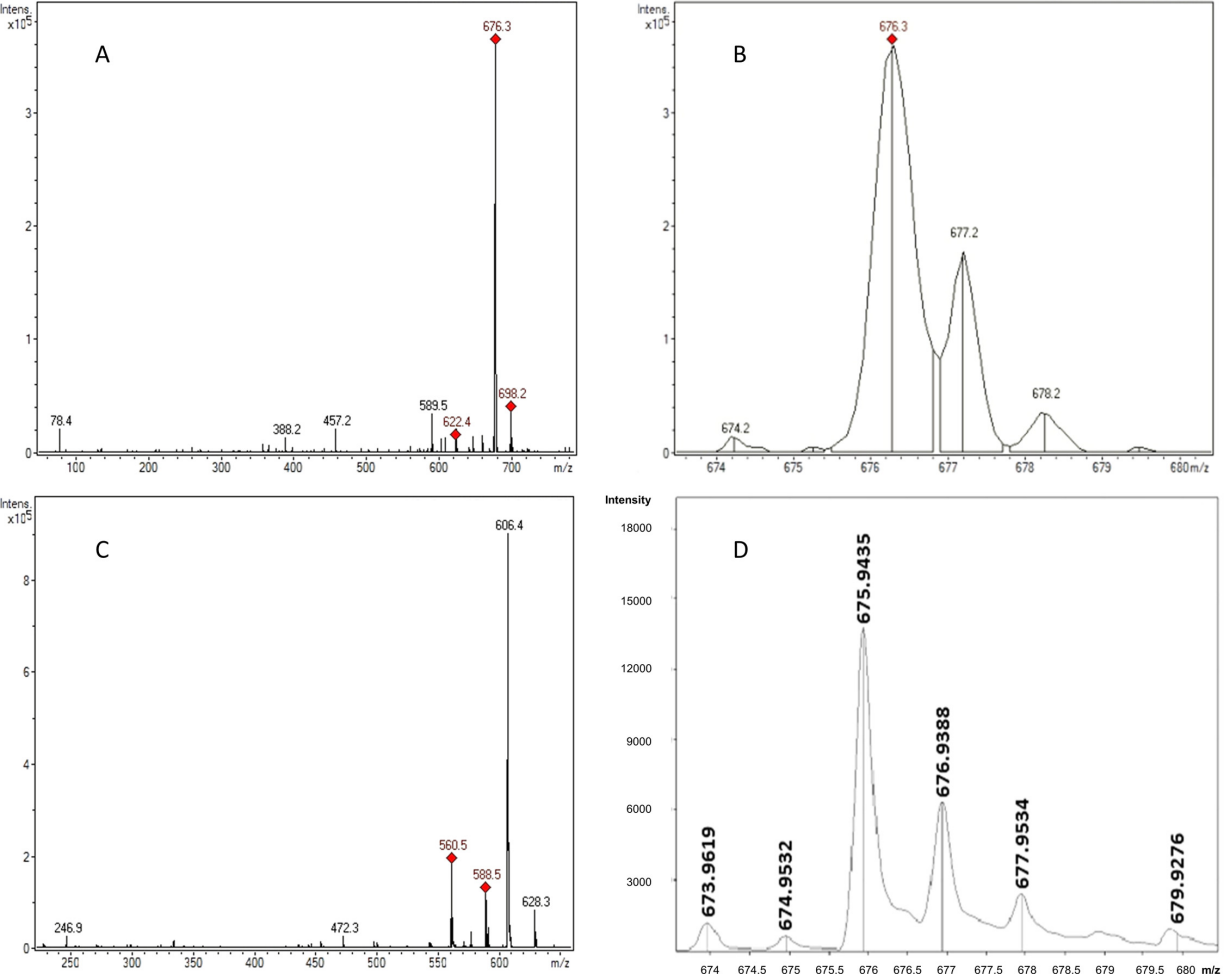
Mass spectrometry of the ferrisiderophore complex. A-C, Siderophore iron complex determined by ESI-MS. A, The molecular peak of the ferrisiderophore complex has a mass of 676.3 Da [M(Fe)+H]^+^. The peak of the siderophore without iron has a molecular mass of 622.4 Da [M)+H]^+^. The peak of 589.5 Da is the fragmentation product [M(Fe)+H-putrescine]^+^. The peak of 698.2 Da is a Na adduct [M(Fe)+Na]^+^. B, Isotopic distribution pattern of the quasimolecular ion of 676.3 Da. The peak of 676.3 Da is the ferrisiderophore complex with most abundant isotopes. The peaks of 674.2 Da, 677.2 Da and 678.2 Da are isotope peaks. C, Fragments of the siderophore iron complex. The peak of 606.4 Da is the fragmentation product formed mainly by the elimination through a McLafferty rearrangement of CH_2_CHCH_2_CH_2_NH_2_. The peaks of 588.5 Da and 560.5 Da are other fragmentation products bound to iron. D, Isotopic distribution pattern of the ferrisiderophore determined by MALDI-TOF mass spectrometry. The peak of 675.9435 Da corresponds to a population of ferrsiderophore complex that contains most abundant carbon^12^, hydrogen^1^, nitrogen^14^, oxygen^16^ and iron^56^ isotopes. The peaks of 676.9388 Da, 677.9534 and 679.9276 Da correspond to populations of complexes that contain lower abundance isotopes in their structure. The peak of 673.9619 Da and 674.9532 Da corresponds to a population of complexes that contain the isotope iron^54^.

To determine the product synthesized by the *mba* gene cluster, the synthesis of a siderophore by strain LB400 was evaluated. Cells were grown in iron-limited medium supplemented with glucose and Orn. To study potential ferrisiderophore complexes, supernatants were treated with iron and subsequently collected, concentrated and subjected to HPLC analysis. HPLC chromatogram of a supernatant from LB400 cells grown under iron-limiting conditions revealed a peak with a retention time of 3.5 min (Fig 6). To determine the structure of the siderophore, HPLC fractions containing the ferrisiderophore complex were collected and subjected to electrospray ionization and mass spectrum analysis (ESI-MS). A quasimolecular ion of 676.3 Da [M(Fe)+H]^+^ with the isotope iron^56^ was identified in the ESI mass spectrum (Fig 7A). The *in silico* analysis predicted that the mass for the non-protonated form of the malleobactin is 622.5526 Da [M] and for the non-protonated form of the ferrimalleobactin complex is 675.2037 Da [M(Fe)]. The presence of iron in this compound was confirmed by the isotopic iron distribution pattern of the quasimolecular ion. The molecular mass of 674.2 Da is the isotopic peak from the siderophore complex with the isotope iron^54^ (Fig 7B). The peptide nature of the compound was confirmed by obtaining peptide fragments when the molecular ion of 676.3 Da was subjected to soft ionization by electrospray. The peak of 589.5 Da is the fragmentation product [M(Fe)+H-putrescine]^+^. The elimination of putrescine (-NHCH_2_CH_2_CH_2_CH_2_NH_2_) from malleobactin in mass spectra has been reported [16]. The fragments of the tetrapeptide bound to iron 606.4 [M(Fe)-70]^+^, 588.5 Da [M(Fe)-88]^+^ and 560.5 Da [M(Fe)-116]^+^ were determined (Fig 7C). The fragments 588.5 Da and 560.5 Da were probably formed by elimination of NHCH_2_CH_2_CH_2_CH_2_NH and CONHCH_2_CH_2_CH_2_CH_2_NH, respectively. The fragment 606.4 Da may be formed mainly by the elimination through a McLafferty rearrangement of CH_2_CHCH_2_CH_2_NH_2_. The presence of iron in the ferrisiderophore complex was confirmed by the isotopic distribution pattern in the 675.9435 Da peak by MALDI-TOF mass spectrometry (Fig 7D). These results confirm the formation of an iron siderophore complex by *B*. *xenovorans* LB400 cultures grown in iron-limited medium and treated with FeCl_3_. The molecular mass of the ferrisiderophore complex matches the theoretically predicted mass for the tetrapeptide bound to iron.

## Discussion

**Fig 8:**
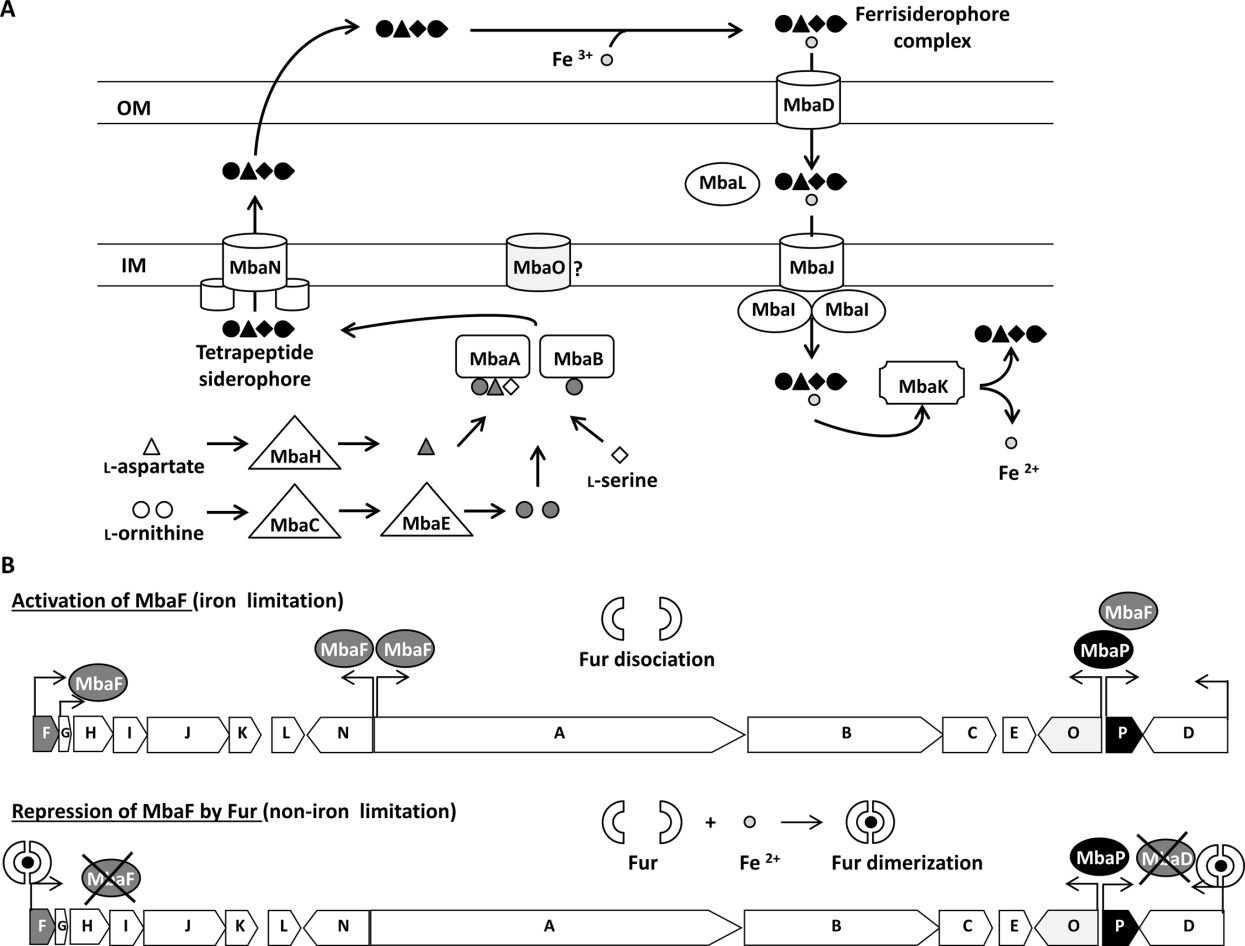
Model for the biosynthesis, transport and regulation of the non-ribosomal peptide siderophore in *B*. *xenovorans* LB400 based on bioinformatic analyses. A, The amino acids are modified by MbaH, MbaC and MbaE enzymes and assembled by MbaA and MbaB NRPS that synthetize the siderophore. The siderophore is exported through MbaN transporter localized in the inner membrane (IM). The siderophore binds iron(III) in the extracellular space, forming the ferrisiderophore complex. The complex is recognized by MbaD in the outer membrane (OM) and imported through the inner membrane by the MbaIJL ABC transporter. From the cytoplasmic ferrisiderophore complex the iron is released by MbaK. MbaO MFS transporter may be involved in siderophore transport, but its function is unknown. B, The transcriptional expression of the *mba* gene cluster from *B*. *xenovorans* LB400 is repressed by iron. In presence of iron, dimerized Fur binds to the Fur box present in the *mbaF* and *mbaD* genes and prevents their transcription. In absence of iron, Fur inactivation released ECF sigma factor binding site of *mbaF* gene, which allows the transcription of the *mbaF* gene. MbaF factor binds to the MbaF boxes in the *mbaG*, *mbaN*, *mbaA* and *mbaP* genes promoters for their expression. The *mbaP* gene transcription is also expressed in iron-rich condition. The LysR-type transcriptional regulator MbaP binds to the LysR binding site present in the *mbaP* and *mbaO* genes allowing their expression.

This is the first report that characterized a siderophore and the NRPS gene cluster associated to its biosynthesis in *B*. *xenovorans* LB400. This NRPS gene cluster comprises 15 coding sequences that are involved in the synthesis and transport of a hydroxamate-type siderophore and shows an unique gene organization. Based mainly on bioinformatic analyses, Fig 8 illustrates a model for the synthesis, transport and regulation of the malleobactin sideophore in *B*. *xenovorans* LB400. The presence of NRPS gene clusters that synthesize hydroxamate-type siderophores have been described in *Burkholderia*. The *orb* gene cluster encoding proteins of the siderophore ornibactin pathway from *B*. *cenocepacia* has been characterized [14]. The *mba* gene clusters from *B*. *pseudomallei* and *B*. *thailandensis* encode enzymes for the synthesis of malleobactins [15–17]. Two novel coding sequences (*mbaO* and *mbaP*) are present in LB400 *mba* gene cluster, which are absent in *B*. *pseudomallei* and *B*. *cenocepacia*. In this study, we also identified by bioinformatic analysis the NRPS *mba* gene cluster from the plant-promoting strain *B*. *phytofirmans* PsJN that contains the *mbaO* and *mbaP* genes. The *mba* gene clusters from strains LB400 and PsJN are highly similar, suggesting a closely related evolution. However, the *mba* gene cluster from strain PsJN possess two additional genes: the *mbaM* gene encoding a protein of unknown function that is also present in the *mba* gene cluster from *B*. *pseudomallei* K96243 [16], and a second *mbaD* gene. *B*. *phytofirmans* PsJN is the only *Burkholderia* strain that possess two *mbaD* genes (Fig 2).

The NRPS gene cluster from strain LB400 allowed the prediction of the siderophore, the linear tetrapeptide hfOrn-D-β-OH-Asp-Ser-hfOrn-1,4-diaminobutane. The supernatant of LB400 cells grown under iron limitation showed a yellow color after incubation with FeCl_3_ and an orange color by the CAS assay, indicating the presence of a siderophore. ESI-MS and MALDI-TOF-MS analyses confirmed the siderophore structure. The presence of iron in the complex and the molecular mass of the iron-siderophore complex (676 Da) strongly support the bioinformaticpredicted siderophore structure. Closely related siderophore structures have been recently reported as malleobactin E and malleobactin B in *B*. *mallei* and *B*. *thailandensis* [16,17]. In strain LB400 the synthesis of a malleobactin isomer that has the formyl group in the nitrogen α of the first Orn could not be excluded. Interestingly, eight different malleobactins were detected in *B*. *thailandensis* [17]. The phylogenetic analyses of specific genes in the NRPS clusters from *Burkholderia* strains differentiate malleobactin-type and ornibactin-type gene clusters. The main structural difference between these two tetrapeptides is the first Orn: hfOrn or hαfOrn in malleobactins and haOrn in ornibactins. Other bacterial siderophores that exhibit hfOrn in their structure are pyoverdin, coelichelin, exochelin and rhodochelin, which are synthesized by *P*. *aeruginosa*, *S*. *coelicolor* [50], *Mycobacterium smegmatis* [51] and *Rhodococcus jostii* [52], respectively. In our study it has been found that malleobactin-type siderophores may be synthesized by several *Burkholderia* strains and a *C*. *fungivorans* strain. A second difference of the malleobactin- and ornibactin-type gene clusters of *Burkholderiales* is the phylogenetic clade distribution of FTs MbaE and PvdF. MbaE-type FTs involved in the production of both hαfOrn and hfOrn for malleobactin-type siderophores, are grouped in a different clade than FTs from the ornibactin (PvdF), pyoverdin (PvdF), coelichelin (CchA), exochelin (FxbA) and rhodochelin (Rft) systems that only produce hfOrn. FTs involved in formylation of primary or secondary amines are clustered in different phylogenetic branches. FTs involved in secondary metabolism such as the FTs mentioned above, possess the catalytic triad Asn106, His108 and Asp144 [53], however, lack the N-terminus for substrate binding and the SLLP motif that participates in the binding of tetrahydrofolate co-substrate present in FTs involved in primary metabolism such as glycinamide ribonucleotide transformylase and methionyl-tRNA_f_^Met^ FT [54]. Based on the phylogenetic analyses of A domains and FTs, as well as the detection of a malleobactin-type compound in *B*. *xenovorans* LB400, a model for the siderophore synthesis is proposed.

Diverse *Burkholderia* strains have multiple iron acquisition mechanisms. Some *Burkholderia* strains (e.g., *B*. *cepacia)* produce more than one siderophore [12,13,15]. Alternative strategies for iron uptake have also been reported [12,55]. *B*. *cenocepacia* and *B*. *pseudomallei* use haemin and ferritin for iron acquisition [56,57]. Recently, a novel siderophore-independent ferric iron-uptake system has been identified in *Burkholderia* strains. The locus Ftr_Bcc_ABCD (*BxeA3149*–*BxeA3152*) from strain LB400 encodes a high affinity ferrous iron transporter [58]. Nevertheless, the *mba* gene cluster is the only LB400 gene cluster that encodes proteins involved in the synthesis of a siderophore.

Iron limitation and the presence of Orn increased LB400 siderophore activity, suggesting a regulated expression of the *mba* genes. RT-PCR results showed that the expression of the *mba* genes from strain LB400 is regulated by iron. Based mainly on bioinformatic analyses and previous studies, we propose a model for regulation of the *mba* gene cluster from strain LB400 (Fig 8). The presence of the *mbaP* gene that encodes a LysR-type transcriptional regulator in the LB400 *mba* cluster and the presence of LysR-type transcriptional regulator binding sites in the promotor regions of the *mbaP* and *mbaO* genes suggest an additional transcriptional regulation. RT-PCR assays showed that the *mbaP* and *mbaO* genes are transcribed in presence of iron-limitation, suggesting that their expression is not repressed by Fur. The transcriptional activation of siderophore biosynthesis genes by LysR-type transcriptional regulators has also been reported in other bacteria. In *P*. *aeruginosa*, under iron limitation the LysR-type regulator PtxR activates through the sigma factor PvdS the transcription of genes associated to pyoverdin biosynthesis. The regulation of *ptxR* gene expression by PvdS is Fur-independent under aerobic conditions [46,59]. Based on bioinformatics analyses we propose that the expression of most of the siderophore biosynthesis, transport and regulation genes is regulated by Fur and/or MbaF (Fig 8), whereas the *mbaP* gene is also autoregulated and *mbaO* gene may be regulated by the transcriptional regulator MbaP.

*B*. *xenovorans* strains have been associated with the rhizosphere [25]. The biosynthesis of a siderophore in *B*. *xenovorans* LB400 suggests that the capture of iron in its rhizosphere ecosystem is relevant for bacterial fitness. Iron could be scarce in soils and, therefore, a limiting nutrient for their bacterial inhabitants. The malleobactin synthesized by *B*. *xenovorans* LB400 may improve its fitness in the rhizosphere and could potentially be used also by plants for iron acquisition [60–62]. Siderophore-producing bacteria may participate also in control of phytopathogens [63–65]. The presence of orthologous *mba* genes in diverse *Burkholderia* species strongly suggests that the production of non-ribosomal peptide siderophores are relevant for *Burkholderia* living in diverse niches such as rhizosphere, soil and (immunocompromised) mammalian organisms. In conclusion, this study describes in the model bacterium *B*. *xenovorans* LB400 a functional and regulated NRP siderophore pathway that synthesized a malleobactin siderophore under iron limitation.

## Supporting Information

S1 Fig Model of the 3D structure of the protein encoded by the LB400 *mbaO* gene and docking with the proposed malleobactin structure. Malleobactin (A) and ferrimalleobactin complex (D) 3D structures were predicted. The binding site for malleobactin 3D structure (B) and ferrimalleobactin 3D structure (E) was predicted using a Docking strategy. The frontal view of the best model of the protein-ligand complexes (C,F) are shown as ribbon representations, where the periplasmic side is located up and the malleobactin and ferrimalleobactin ligands surfaces are highlighted in blue. The cytoplasmic (G) and periplasmic (H) sides, and the volume (I) of the protein ligand complex are shown.
(TIF)

S1 Table Primers sets designed and used in this study. (DOC)
