# Supplementary figures and images for: Genetic and Functional Analysis of the Biosynthesis of a Non-Ribosomal Peptide Siderophore in Burkholderia xenovorans LB400
Source: PLoS One. 2016 Mar 10;11(3):e0151273. doi: 10.1371/journal.pone.0151273 (PMC4786211; doi:10.1371/journal.pone.0151273)

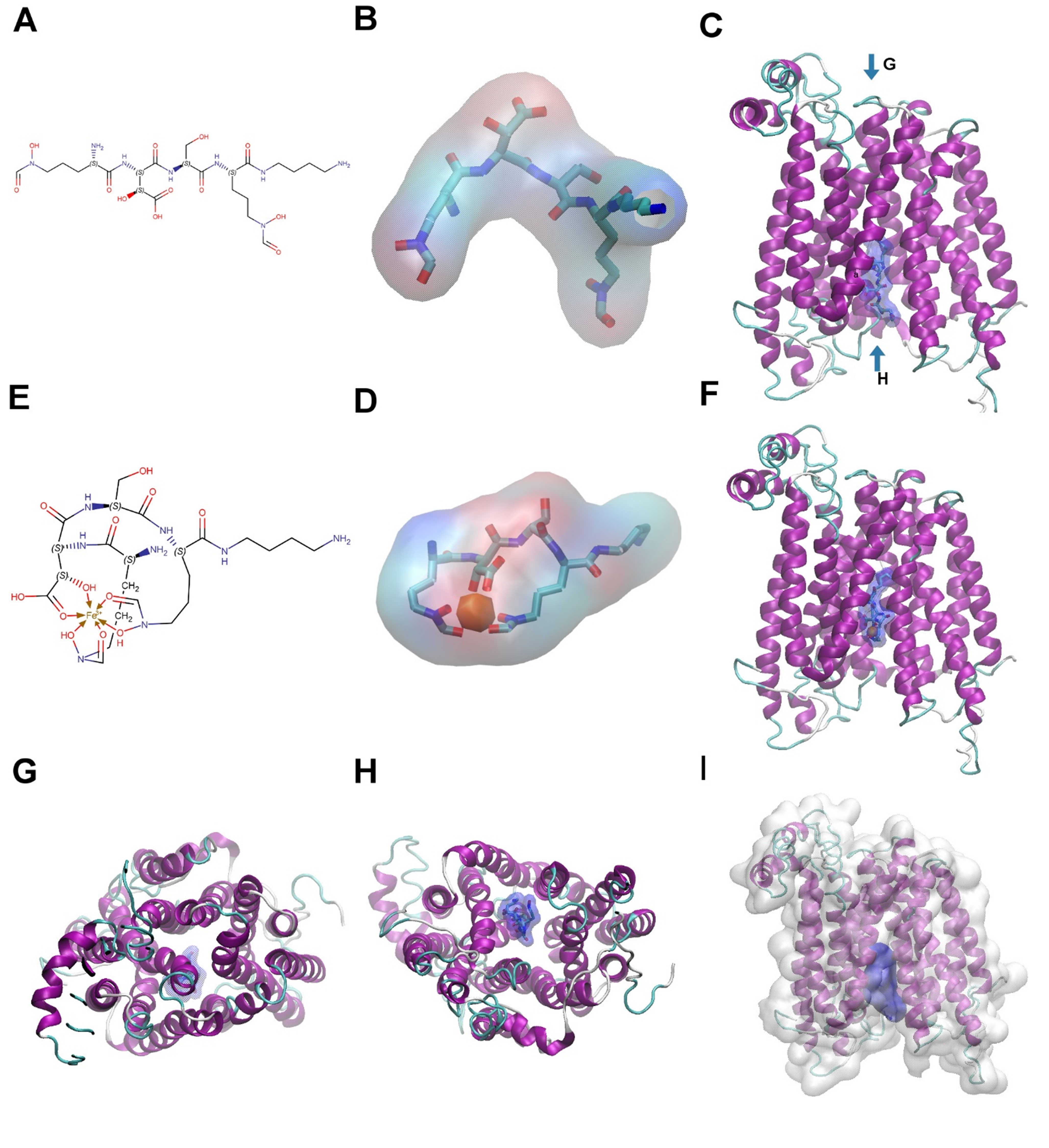

Supplement: S1 Fig — Malleobactin (A) and ferrimalleobactin complex (D) 3D structures were predicted. The binding site for malleobactin 3D structure (B) and ferrimalleobactin 3D structure (E) was predicted using a Docking strategy. The frontal view of the best model of the protein-ligand complexes (C,F) are shown as ribbon representations, where the periplasmic side is located up and the malleobactin and ferrimalleobactin ligands surfaces are highlighted in blue. The cytoplasmic (G) and periplasmic (H) sides, and the volume (I) of the protein ligand complex are shown. (TIF) [file pone.0151273.s001.tif]
