# Supplementary material for: Genetic and Functional Analysis of the Biosynthesis of a Non-Ribosomal Peptide Siderophore in Burkholderia xenovorans LB400
Source: PLoS One. 2016 Mar 10;11(3):e0151273. doi: 10.1371/journal.pone.0151273 (PMC4786211; doi:10.1371/journal.pone.0151273)
Supplement: S1 Table — (DOC) [file pone.0151273.s002.doc]

**S1 Table.** **Primers sets designed and used in this study**

| **Genic or intergenic region** | **Name** | **Sequence (5´-3´)** |
| --- | --- | --- |
| *mbaF* promoter | pFf | CGCCGATACTCGGAGTGTTA |
| *mbaF* promoter | pFr | CGAGCACCTCAGCCATAGAT |
| *mbaF-mbaG* | F-Gf | CCTGGAAAACACCTATCACG |
| *mbaF-mbaG* | F-Gr | ACTGCTCTTCGTCGTTGATG |
| *mbaG-mbaH* | G-Hf | CAAGCACAGCAGAGTTCCAG |
| *mbaG-mbaH* | G-Hr | CATTCTCGCGTGTACGATTG |
| *mbaH-mbaI* | H-If | TCAGCAGCGTATCGTGTTTC |
| *mbaH-mbaI* | H-Ir | AGTCCCGAAGGAATCTGGTT |
| *mbaI-mbaJ* | I-Jf | CCTACAGCGACGACATTGTG |
| *mbaI-mbaJ* | I-Jr | GCGTCAGCGACTGGAATAAG |
| *mbaJ-mbaK* | J-Kf | CGGTCGGTTTTATCGGTTTG |
| *mbaJ-mbaK* | J-Kr | AGTTCGCTGACGGGAATCAC |
| *mbaK-mbaL* | K-Lf | GGCAATCTGCTGGATTACCT |
| *mbaK-mbaL* | K-Lr | GCATACGAACCGGAGAAAAC |
| *mbaL-mbaN* | L-Nf | GCCCGAACTTTACCGAAGAC |
| *mbaL-mbaN* | L-Nr | GTCACGACGACCGCTATTT |
| *mbaN-mbaA* | N-Af | TTGGTGGGAGAGGAGGAAGA |
| *mbaN-mbaA* | N-Ar | CTTCAATACGGCAAGCAACG |
| *mbaA-mbaB* | A-Bf | GACACACAAACGCTGCTCA |
| *mbaA-mbaB* | A-Br | TGCTGGGTTGTCTCAGTACG |
| *mbaB-mbaC* | B-Cf | TGGAGATTGCCGACGTGTT |
| *mbaB-mbaC* | B-Cr | ACGCCAGGTAATCGTGGAAC |
| *mbaC-mbaE* | C-Ef | GCTGTTGCGAAGACAGTCAC |
| *mbaC-mbaE* | C-Er | ATGGCGAGTAGCAGATCGTT |
| *mbaE-mbaO* | E-Of | CGTGGCAGGAAGGTAGTCAA |
| *mbaE-mbaO* | E-Or | GACTCGTCTGGAAGCATCGT |
| *mbaO-mbaP* | O-Pf | CATCAGAACCGTTGTTACCG |
| *mbaO-mbaP* | O-Pr | TCGAGCCTCACGATCACTT |
| *mbaP-mbaQ* | P-Qf | CCGCTATCTACCGCTGAAAA |
| *mbaP-mbaQ* | P-Qr | ACGTGGACAACATCTTCGTG |
| *mbaQ* - B0532 | Q-B0532f | GAAGAGGACAGCGTGGAAAG |
| *mbaQ* - B0532 | Q-B0532r | GATGATCTTCGGCGATGTG |
| *mbaN* | Nf | GAAGACGGCACCATTGAACT |
| *mbaN* | Nr | AAATAGCGGTCGTCGTGACT |
| *mbaO* | Of | AGTTGGCAATGGGTCTTCTG |
| *mbaO* | Or | GAAAACTGCGGAATCCTGAA |
| *mbaP* | Pf | GACGGGCAGGTCTACTACGA |
| *mbaP* | Pr | CACGAAATCGAAACCCTCTT |
| 16S rRNA | 27F* | AGAGTTTGATCMTGGCTCAG |
| 16S rRNA | 1492R* | TACGGYTACCTTGTTACGACT |

* Standard primer used for 16S rRNA gene amplification.
